# The CGAS-STING1 Pathway as a Mediator of Innate Immune Response in Cardiovascular Disease

**DOI:** 10.1016/j.jacasi.2025.01.005

**Published:** 2025-03-18

**Authors:** Leila Rouhi, Priyatansh Gurha, Ali J. Marian

**Affiliations:** Center for Cardiovascular Genetic Studies, Institute of Molecular Medicine, The University of Texas Health Science Center, Houston, Texas, USA

**Keywords:** cardiomyopathy, CGAS, cytosolic DNA, DNA damage response, heart failure, immunity

## Abstract

The innate immune response, a rapid and cell-autonomous response of the cell to the pathogens, recognizes the external as well as the internal pathogens, such as self-DNA, released from the damaged cells. The response activates a set of molecules that induce the expression of proinflammatory cytokines and chemokines and leads to inflammation, fibrosis, and cell death. The innate immune response comprised of DNA-sensing protein cyclic guanosine monophosphate–adenosine monophosphate synthase (CGAS) and its downstream molecules, the stimulator of interferon genes 1 (STING1), TANK-binding kinase 1 (TBK1), interferon regulatory factor 3 (IRF3), and nuclear factor kappa B (NFκB), are activated in several cardiovascular diseases, including hereditary cardiomyopathies, myocardial infarction, hypertension, atherosclerosis, and aortic aneurysm. The genetic deletion of key molecules in this pathway, such as CGAS, STING1, and interferon regulatory factor 3, affords salubrious effects, including improving survival and cardiac dysfunction, rendering the CGAS-STING1 pathway an attractive therapeutic target in cardiovascular disease.

## Highlights

• The self-DNA is released in pathological conditions.
• The CDSP pathway is activated in response to self-DNA.
• The activated CDSP pathway provokes inflammation, cell death, fibrosis, and organ dysfunction.
• The CDSP pathway is a therapeutic target in cardiovascular diseases.

Innate immunity, as opposed to acquired or adaptive immunity, is the intrinsic, constitutive, rapid, and protective response of the organism designed to destroy internal or external pathogens. Innate immunity encompasses the physical and mechanical barriers and extends to the cellular and molecular defenses, including the activation of the macrophages and the expression of the proinflammatory cytokines. The key component of innate immunity is the pattern recognition receptors (PRRs), such as toll-like receptors (TLRs), which recognize the pathogen-associated molecular patterns released from the pathogens or the damage-associated molecular patterns (DAMPs) released from the damaged cells. Pathogen-associated molecular patterns and DAMPs, which comprise cellular proteins and nucleic acids, are sensed by the components of the innate immune systems and activate the inflammasome and the expression of the proinflammatory cytokines and chemokines, among others. The responses induce inflammation, fibrosis, cell death, and cell cycle arrest, with the main objective of arresting replication of the invading pathogen and imposing death to the pathogen and/or the cell carrying the pathogen. Activation of innate immunity is likely the underpinning mechanism of the several autoimmune disorders and low-grade inflammation that is present in aging and various cardiovascular pathologies, ranging from atherosclerosis to heart failure.

Among the numerous components of innate immunity are about a dozen proteins that recognize cytosolic DNA, including the self-DNA. These proteins, which we refer to as the cytosolic DNA-sensing proteins (CDSPs), have emerged as the key molecules in innate immune response and are major mediators of the interferon and inflammatory responses of the cells to the internal and external DNA. Notable among the CDSPs is the cyclic guanosine monophosphate–adenosine monophosphate synthase (CGAS), which is among the best-studied mediators of the cellular response to the cytosolic DNA. The significance of the CDSP proteins in innate immunity was recently recognized by awarding the 2024 Albert Lasker Basic Medical Research Award to Dr. Zhijian “James” Chen for his discovery of the cytosolic DNA-sensing protein CGAS, which helped to solve the mystery of how the cell senses the cytosolic self and exogenous DNA to initiate an innate immune response. In this review, we focus on the biological and clinical roles of CGAS and selected proteins in the CDSP pathway in the cardiovascular system.

## Cytosolic DNA-Sensing Proteins

The cell is equipped with a set of PRRs that recognize the invading viral and bacterial DNA and proteins and provoke an innate immune response as a defense mechanism. The cell defense tools comprise about a dozen CDSPs that recognize the DNA based on the length but not the sequence of the nucleotides. Consequently, the CDSPs do not discern the self from foreign DNA, and any cytosolic DNA, depending on its length and concentration regardless of its sequence, is recognized by the CDSPs. The cytosolic self-DNA often originates from the mitochondrial DNA (mtDNA), because mitochondrial dysfunction is ubiquitous in the pathological states and results in the release of mtDNA to the cytosol and consequent activation of an inflammatory response.^1^^,^^2^ The innate immune response to mtDNA is the underpinning cause of several autoimmune diseases in humans, such as systemic lupus erythematosus, rheumatoid arthritis, and inflammatory bowel disease.^2^ Likewise, increased internal genomic stressors in the pathological states, such as alkylating agents or cross-linkers, by damaging the nDNA could lead to the release of the nDNA into the cytosol and its recognition by the CDSPs.^3^^,^^4^ The cytosolic self-DNA, whether mtDNA or nDNA, instigates an innate immune response by activating the CDSPs and leading to type I interferon response, inflammation, and cell death.

The CDSPs show partial tissue-specificity in their expression patterns, but all share the common function of recognizing the cytosolic DNA, despite their considerable structural diversity.^5^ The prototypic example of the CDSPs is the CGAS protein, which is coded by the *Mb21d1* gene and recognizes mainly double-stranded cytosolic DNA.^6^, ^7^, ^8^ The affinity of CGAS for DNA is dependent on the length as well as the concentration of DNA. It is most sensitive to the longer DNA fragments but can also detect DNA fragments as small as 25 nucleotides in length.^9^ Its activity, however, is the highest for longer DNA fragments because CGAS uses the DNA as a template to cluster in a ladder-like manner around the DNA and form condensates.^9^^,^^10^

Other CDSPs, such as the Z-DNA binding protein 1 (ZBP1), recognize both the B- and the Z-forms of DNA; the former is the right-handed helix (Watson-Crick helix), and the latter is a left-handed helix. Other notable members of CDSPs are absent in melanoma 2 (AIM2), which activates the inflammasome; NACHT Domain-, Leucine-Rich Repeat-, and PYD-Containing Protein 3 (NLRP3), Toll-Like Receptor 9 (TLR9), interferon gamma-induced protein 16 (IFI16), and DEAD-Box Helicase 41 (DDX41).^5^ The diversity of the CDSPs signifies the crucial role of these molecules in defending against external sources of DNA and in mediating the innate immune response by sensing the external and self-DNA in various pathological states.

The CDSPs often form tertiary structures upon dimerization or oligomerization, which influence their functions. For example, CGAS and ZBP1 form dimers upon sensing the cytosolic DNA, whereas others such as IFI16 and AIM2 oligomerize.^5^^,^^9^ CGAS upon initial dimerization forms a ladder-like arrangement over the length of the DNA and clusters into molecular condensates rich in CGAS with an increased enzymatic activity.^10^ The CDSPs, upon binding to the cytosolic DNA, directly or indirectly activate their adaptor target proteins, such as the stimulator of interferon genes 1 (STING1, aka, TMEM173), and induce the downstream type I interferon response and the expression of the proinflammatory cytokines, among others.

## Biological Functions of the CGAS-STING1 Pathway

**Figure 1:**
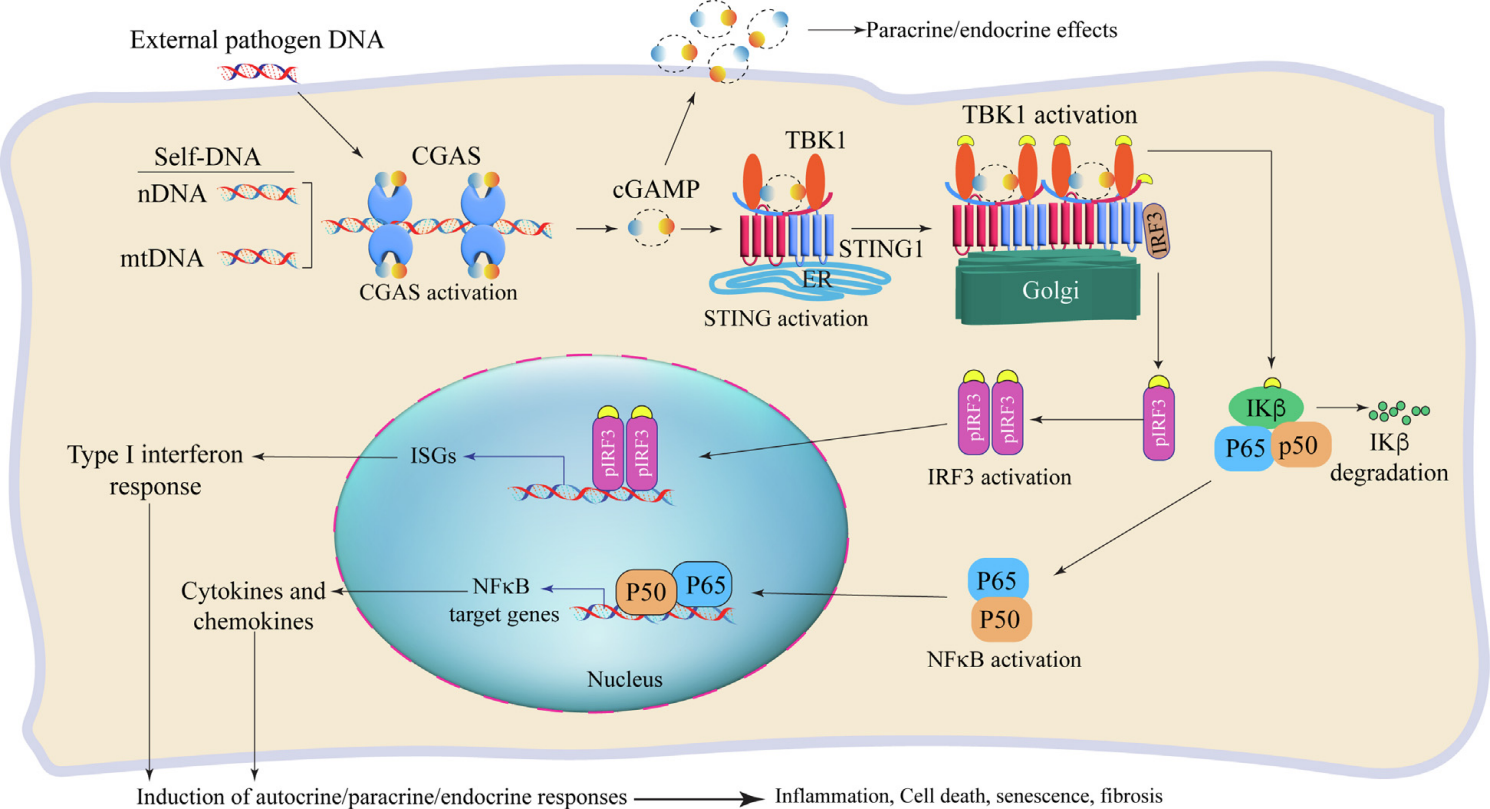
The Cytosolic DNA-Sensing Protein Pathway The cytosolic DNA is sensed by cyclic guanosine monophosphate–adenosine monophosphate synthase (CGAS), which forms CGAS oligomers and catalyzes the synthesis of cyclic GMP-AMP (cGAMP). The latter binds to the stimulator of interferon genes 1 (STING1) and TANK-binding kinase 1 (TBK1) complex, leading to oligomerization and translocation of STING1 to Golgi apparatus and subsequent phosphorylation and activation of TBK1. The activated TBK1 phosphorylates interferon regulatory factor 3 (IRF3), leading to its translocation into the nucleus and induction of expression of the interferon-stimulated genes (ISGs). The activated TBK1 also activates of the nuclear factor kappa B (NFκB) by phosphorylating the inhibitor of the nuclear factor κB (IKβ) and its degradation. The activated components of the NFκB, namely P50 and P65, translocate into the nucleus and induce the expression of the proinflammatory cytokines/chemokines. The end phenotype is cell death, senescence, fibrosis, and inflammation, organ dysfunction, and premature death.

CGAS, discovered in 2013, is activated upon binding to the cytosolic DNA in a length-dependent (typically >25 bp) but not sequence-specific manner.^11^^,^^12^ In addition to the bacterial and viral DNA and free self-DNA, CGAS is also activated by the DNA in cytosolic micronuclei, chromatin fragments, and extrachromosomal telomere DNA.^13^ CGAS upon binding to cytosolic DNA forms dimers, which are arrayed along the length of the cytosolic DNA and form mesh-like complex molecular condensates.^9^^,^^10^ The active CGAS utilizes adenosine monophosphate (AMP) and guanosine monophosphate (GMP) to catalyze the synthesis of cyclic GMP-AMP (cGAMP) that can act in an autocrine or paracrine manner (Figure 1).^11^ The cGAMP binds and activates STING1, which is a hub for several CDSPs, including IFI16 and DDX41, in addition to CGAS.^14^^,^^15^

CGAS structure and function are affected by extensive post-translational modifications.^12^ For example, monoubiquitinylation of CGAS by TRIM56 activates, whereas phosphorylation of CGAS by AKT at Ser291 (Ser305 in human CGAS) inactivates its enzymatic activity in generating cGAMP.^16^^,^^17^ CGAS is also regulated by several cofactor proteins that bind to CGAS and affect its dimerization and, hence, its enzymatic activity.^12^

In addition to its localization to the cytosol where it generates cGAMP, CGAS also localizes to the nucleus and has been detected in the cytoplasmic membrane, micronuclei, and lysosomes.^18^, ^19^, ^20^, ^21^ The nuclear CGAS does not have a DNA-sensing function because it is tightly tethered to chromatin and is maintained in the monomeric form by histone H2A and H2B.^20^^,^^22^ This is consistent with the essential role of dimerization in the enzymatic activity of CGAS. The function of nuclear CGAS is not well known; suffice it to state that nuclear CGAS is implicated in binding to the DNA damage repair proteins, suppressing the repair process, and slowing the replication fork, which may lead to genomic instability.^22^, ^23^, ^24^

The best-known function of the cytosolic CGAS is to activate STING1 through generating cGAMP. STING1 is a dimeric protein that is mainly located at the endoplasmic reticulum (ER) in association with TANK-binding kinase 1 (TBK1) molecules (Figure 1).^25^ Upon binding of cGAMP, STING1 undergoes conformational changes that promote its oligomerization and translocation of the STING1, in complex with TBK1, from the ER to ER–Golgi intermediate compartment, where TBK1 is activated by auto-phosphorylation.^9^ The activated TBK1 phosphorylates STING1, and the complex serves as a platform to recruit and phosphorylate interferon regulatory factor 3 (IRF3). The phospho-IRF dimerizes and translocates into the nucleus, where it induces the expression of genes involved in type I interferon response (Figure 1).^9^^,^^26^^,^^27^ In addition to the activation of IRF3, the STING1/TBK1 complex also activates the nuclear factor kappa-B (NFκB) pathway, presumably by targeting the inhibitors of nuclear factor kappa-B kinase beta (IKKβ) for phosphorylation and degradation.^28^^,^^29^ Degradation of IKKβ frees the NFκB from the inhibitor and leads to increased nuclear localization of the core effectors P50 and p65.^28^^,^^29^ The activation of IRF3 and NFκB pathways leads to the expression of the type I interferon response genes and the proinflammatory cytokines and chemokines, which induce sterile inflammation, fibrosis, cell death, and cell senescence.

STING1 is a multifunctional protein and is also involved in autophagy and lysosomal-dependent cell death, which are not well understood but are partly independent of TBK1 and IRF3 activation.^21^^,^^30^ Genetic mutations also provide additional clues to the biological functions of STING1 as the gain-of-function mutations in the *TMEM173* gene, encoding STING1, cause “STING-associated vasculopathy with onset in infancy,” which is an autoinflammatory disease and in part independent of activation of the IRF3.^31^^,^^32^ In contrast, the loss-of-function mutations in the *TMEM173* gene are often observed in tumor cells and lead to a suppressed immune response enabling the cancer cells to avoid the immune response.^33^

## Activation of the CGAS-STING1 Pathway in Cardiovascular Disease

Since the discovery of CGAS about a decade ago, there has been considerable interest in the biological role of this CDSP in various conditions including cardiovascular diseases.^11^ Considering that cell death is common in cardiovascular diseases and because cell death releases DAMPs, which activates the PRRs, activation of the CGAS-STING1 pathway in cardiovascular disease is not unexpected. The existing data supports this notion by documenting the activation of the CGAS-STING1 as well as the nuclear component of the DNA damage response (DDR) pathway, namely the ataxia telangiectasia mutated-tumor protein 53 (ATM-TP53) pathway in numerous cardiovascular diseases (Central Illustration).^34^, ^35^, ^36^, ^37^, ^38^, ^39^, ^40^, ^41^, ^42^, ^43^, ^44^

**Central Illustration:**
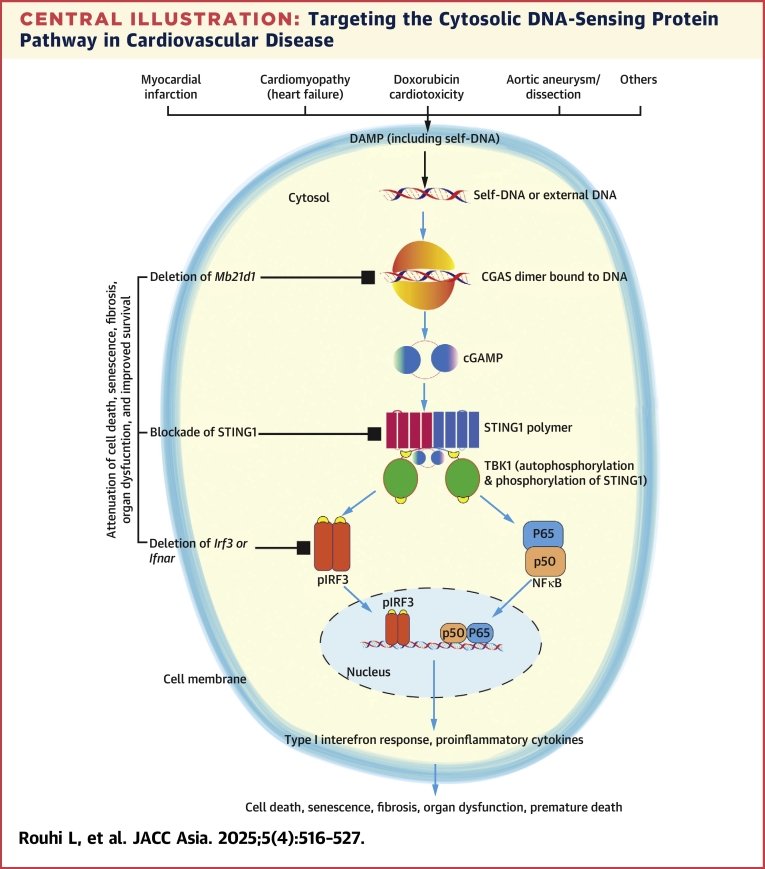
Targeting the Cytosolic DNA-Sensing Protein Pathway in Cardiovascular Disease Cell damage releases damage-associated molecular pattern (DAMP), including self-DNA, which is sensed by cytosolic DNA-sensing proteins such as cyclic guanosine monophosphate–adenosine monophosphate synthase (CGAS), which dimerizes and synthesizes cyclic guanosine monophosphate adenosine monophosphate (cGAMP), which binds to stimulator of interferon genes 1 (STING1), leading to its oligomerization and binding to TANK-binding kinase 1 (TBK1). The latter autophosphorylates and phosphorylates STING1, as well as the downstream effector interferon regulatory factor 3 (IRF3). In addition, phosphorylated TBK1 also activated nuclear factor kappa B (NFκB) components P50 and P65. Phospho-IRF3, P50, and P65 translocate into the nucleus and induce type I interferon response and the expression of proinflammatory cytokines/chemokines. Expression of these molecules causes cell death, senescence, fibrosis, organ dysfunction, and premature death. Genetic blockade of Mab21 Domain Containing Protein 1 (*Mb21d1)* gene encoding CGAS, *Sting1*, *Irf3*, or interferon α and β receptors (IFNAR) imparts salubrious effects by improving cell death, fibrosis, organ dysfunction, and survival.

### Myocardial infarction and ischemia/reperfusion injury

Cell death is a prominent feature of myocardial ischemic injury. It leads to the release of DAMPs, including self-DNA (both nDNA and mtDNA), and activation of the PRRs, which provoke an innate immune response. Not surprisingly, therefore, the initial evidence of the activation of the CGAS-STING1 pathway in cardiovascular disease was observed in the setting of myocardial infarction.^35^^,^^45^ Accordingly, permanent coronary ligation in mice, a model of myocardial infarction, by releasing the self-DNA activates the CGAS-STING1 pathways, provoked phosphorylation of IRF3 and induced expression of the type I interferon response genes.^35^^,^^45^ Consistent with the pathogenic role of activation of the CGAS-STING1 pathway, deletion of the *Mb21d1*, *Sting1*, or *Irf3* gene abrogated the induction of the interferon 1 response and improved survival and cardiac remodeling in a mouse model of myocardial infarction.^35^^,^^45^ Notably, however, the deletion of the *Irf3* or *Ifnar* gene, the latter encoding interferon α and β receptors, completely prevented the premature death, whereas the deletion of the *Mb21d1* gene only partially rescued the mortality and the deletion of the *Sting1* gene had no effect.^35^^,^^46^ The complete rescue of postmyocardial infarction mortality upon deletion of *Irf3* or *Ifar* is rather unexpected given the multifarity of the involved mechanism in postmyocardial infarction death and the partial role of the innate immune response. Likewise, pharmacological inhibition of STING1 in a mouse model of ischemia/reperfusion injury improved left ventricular remodeling and systolic function, whereas the intervention had no effect when the left anterior descending coronary artery was permanently ligated.^47^

Multiple cell types seem to contribute to the activation of the type I interferon response in the setting of myocardial injury, including a subset of cardiac macrophages, cardiac fibroblasts, splenic dendritic cells, and a subset of cardiac myocytes located at the border zone of myocardial infarction.^34^^,^^35^^,^^46^ Activation of the CGAS promotes macrophage transformation, whereas loss-of-function of CGAS provokes a reparative phenotype and enhances myocardial repair in the setting of coronary ligation in a mouse model of myocardial infarction.^45^ Moreover, activation of the CDSP pathway is also implicated in the activation of platelets in patients with acute ST-segment elevation myocardial infarction and thrombus formation.^48^ Conversely, depletion of CGAS in platelets ameliorates myocardial ischemia/reperfusion injury and improves cardiac function.^48^

### Hereditary cardiomyopathies

Dysregulation of mitochondrial dynamics and impaired oxidative phosphorylation are common features of myocardial diseases, including cardiac hypertrophy and failure.^49^ Consequently, mitochondrial damage leads to the release of the mtDNA into the cytosol, which is sensed by the CDSPs such as CGAS, ZBP1, and TLR9, leading to the induction of expression of the type I interferon response genes.^37^^,^^50^ Likewise, the presence of significant nDNA damage, such as double-stranded breaks (DSBs), resulting from oxidative and metabolic stresses leads to the release of the nDNA into the cytosol and activation of the CDSP pathway.^43^^,^^51^ The cytosolic release of the nDNA is particularly prominent in conditions where the nuclear membrane is defective, such as in laminopathies.^43^^,^^51^^,^^52^

In support of this notion, expression of the CDSPs, such as CGAS and ZBP1, and activation of the downstream molecular cascade has been demonstrated in hereditary forms of cardiomyopathy caused by defined mutations in humans and mouse models.^39^, ^40^, ^41^^,^^51^^,^^53^ Likewise, activation of the nuclear component of the DDR pathway, comprised of the ATM-TP53 axis, has been detected in human hearts and mouse models of heart failure, including hereditary cardiomyopathies.^40^^,^^43^ Moreover, an increased expression level of phospho-H2A.X variant histone (pH2AFX), a marker of DSBs, in cardiac myocytes has been reported in a mouse model of lamin A (LMNA), TMEM43, and plakophilin 2 cardiomyopathy.^41^, ^42^, ^43^^,^^54^ Collectively, the data support the presence of DNA damage, including strand breaks in cardiomyopathies not only in cardiac myocytes but also in cardiac fibroblasts in multiple genetic models and human hearts. These data exclude model-specific findings and support the generalizability of the activation of the DDR pathways in heart failure, albeit in a limited number of the genetic mutations.

The specific types of DNA lesions in heart failure have not been systematically investigated; however, the existing data support increased oxidative DNA damage, indicated by increased levels of 8-hydroxyl-2′-deoxyguanosine (8-OH-dG), malondialdehyde, and 4-hydroxy-2-nonenal in the heart as well as increased prevalence of DSBs.^51^^,^^55^^,^^56^ Genome-wide analysis of the DSBs in the cardiac myocytes in a mouse model of LMNA cardiomyopathy suggested an increased number of the DSBs and their coupling to transcription.^51^ The latter is based on the observation that DSBs are predominantly localized to the vicinity of the transcription start sites and are more prevalent at the genomic sites with increased transcript levels, as opposed to those with low levels of gene expression.^51^ The coupling of DSBs to transcription is in agreement with the lower prevalence of DSBs at the lamin-associated domains, which are the sites of suppressed transcription as opposed to the genomic regions that are outside of the lamin-associated domains, which have 16-fold higher transcript levels and a 5-time higher prevalence of DSBs.^51^^,^^57^^,^^58^

### Cardiac hypertrophy and failure (other than hereditary cardiomyopathies)

Both mtDNA and nDNA are implicated in the activation of the innate immune response in cardiac hypertrophy and heart failure.^50^^,^^59^ Mitochondrial damage in heart failure and pressure overload-induced cardiac hypertrophy and failure leads to the cytosolic release of mtDNA, which if not degraded through lysosomal mechanisms, activates the CDSP pathway and leads to myocardial inflammation.^50^ Failure to degrade the cytosolic DNA in mice lacking lysosomal DNASE2 enzyme (*Dnas2a* null mice), leads to excess mortality, cardiac dilatation and dysfunction, and myocardial fibrosis in conjunction with a proinflammatory molecular phenotype in response to pressure-overload–induced cardiac hypertrophy.^50^

The CDSP pathway is also activated and pathogenic in pressure-overload–induced cardiac hypertrophy and failure, including in cardiac hypertrophy associated with chronic kidney disease.^38^^,^^59^ Knock-down of CGAS using adeno-associated viruses, serotype 9 expressing short hairpin RNA (shRNA) attenuates cardiac hypertrophy, apoptosis, and fibrosis and improves survival in mice subjected to transaortic constriction.^38^ Likewise, deletion of the *Sting1* gene in cardiac myocytes reduced cardiac hypertrophy in mice with chronic kidney disease, independent of pressure overload.^59^ Finally, in human patients with atrial fibrillation who subsequently develop heart failure, serum cGAMP concentration, a product of CGAS activity, was higher than in those who do not develop heart failure.^60^ The increase might be secondary to heart failure rather than a primary causal factor.

The cardiotoxicity of anthracyclines in part has been attributed to their role in activating the CDSP pathway through multiple mechanisms, including the release of the nuclear and mitochondrial DNA as well as the Z form of DNA.^36^^,^^37^ These chemotherapeutic agents are known to intercalate into DNA strands and block topoisomerase II processivity, leading to the accumulation of DSBs and activation of the DDR pathways.^61^ Likewise, anthracyclines, including doxorubicin, are toxic to mitochondria and cause significant oxidative stress, mitochondrial damage, and the release of the mtDNA to the cytosol and consequent activation of the CDSP pathway in cardiac myocytes.^3^^,^^37^ In addition to cardiac myocytes, treatment with doxorubicin also activates the CDSP pathway in the endothelial cells and leads to endothelial dysfunction.^36^ Deletion of the *Sting1* gene in the endothelial cells attenuates the cardiotoxicity of doxorubicin.^36^ Likewise, pharmacological inhibition of TBK1, a downstream protein of the CDSP pathway, also attenuates the cardiotoxicity of doxorubicin.^36^ In addition to the CGAS-STING1 axis, the Z-DNA binding protein 1, which is up-regulated in mouse models of heart failure and promotes PANoptosis, is also implicated in the cardiotoxicity of doxorubicin.^37^^,^^53^ Accordingly, deletion of *Zbp1, Sting1,* or *Ifnar* attenuates cardiotoxicity of doxorubicin.^37^ Furthermore, myocarditis associated with the immune checkpoint inhibitors also entails activation of the CDPS pathway, in part because of gasdermin-E–mediated pyroptosis and the release of mtDNA.^62^ Thus, multiple mechanisms operate in heart failure to activate the DDR pathways, including the release of the nDNA and mtDNA into the cytosol.

### Systemic arterial hypertension and other vascular diseases

The CDSP is activated in ischemic heart disease, and expression of senescence-associated secretory phenotype is implicated in the pathogenesis of atherosclerosis.^35^^,^^45^^,^^63^ As noted earlier, the cardiotoxicity of the doxorubicin has been attributed, in part, to the activation of the CGAS-STING1 pathway in the endothelial cells.^36^ Activation of CGAS, increased generation of cGAMP, and activation of cGMP-dependent protein kinase in vascular endothelium are also implicated in the pathogenesis of sepsis-induced hypotension.^64^ The cGAMP generated by the CGAS activity in the vascular endothelial cells through a paracrine mechanism activates the cGMP-dependent protein kinase 1, which provokes vasodilation and lowers blood pressure.^64^ In addition to its effects on the vascular system, the CGAS-STING1 pathway also affects blood pressure through the hypothalamic paraventricular nucleus.^65^ In an experimental model of hypertension induced upon infusion of angiotensin II, the CGAS is activated in the hypothalamic paraventricular nucleus, where it promotes neuro-inflammation and increased sympathetic activity.^65^ Genetic and pharmacological inhibition of CGAS lowers blood pressure by attenuating neuroinflammation and reducing sympathetic tone.^65^ Infusion of an inhibitor of CGAS to the hypothalamic cistern also reduced cardiac hypertrophy and myocardial fibrosis and improved cardiac function in an experimental mouse model of hypertension.^65^ In agreement with the role of the CGAS-STING1 pathway in the pathogenesis of vascular disease, deletion of the *Mb21d1* or *Irf3* gene attenuates cellular senescence and reduces the expression levels of the proinflammatory IL6 in mice.^63^

Increased cytosolic nDNA and mtDNA along with the activation of the CDSP pathway also have been detected in the smooth muscle cells and macrophages in the aortic tissues of human patients with ascending thoracic aortic aneurysm and dissection.^44^ In a mouse model of aortopathy, systemic inactivation of *Sting1* gene, genetically and pharmacologically, afforded partial protective effects against the development of aortic aneurysm and dissection.^44^ Likewise, the CDSP pathway is activated in a subset of monocytes and macrophages in a mouse model of aortic aneurysm, and deletion of the *Sting1* gene in the myeloid cells reduces the development, progression, and rupture of adnominal aortic aneurysm in mice.^66^

### Cardiac arrhythmias

Evidence of DNA damage and activation of the nuclear and cytosolic components of DDR originates from observational studies in human patients with atrial fibrillation, partly because of the availability of the atrial tissue, which is excised during surgical procedures. The data suggests increased levels of markers of DNA oxidation such as 8-OH-dG and DNA strand break markers such as pH2AFX, tumor protein 53 binding protein 1 (TP53BP1), and PARP1 in human atrial tissues in patients with persistent atrial fibrillation.^67^, ^68^, ^69^ Likewise, an increased level of poly(ADP)-ribose polymerase 1 (PARP1), which senses the DNA strand breaks and recruits the repair proteins to the break sites, and depletion of nicotinamide adenine dinucleotide are implicated in the pathogenesis of atrial fibrillation.^67^, ^68^, ^69^ Furthermore, increased serum concentration of cGAMP in patients with atrial fibrillation is associated with the occurrence of heart failure.^60^ Whether these findings are secondary to hemodynamic stress or atrial fibrillation or are the primary driver of atrial fibrillation remains to be substantiated. Nevertheless, the data suggests the contribution of DNA damage to the pathogenesis of atrial fibrillation.^68^

## Therapeutic Targeting of the CGAS-STING1 Pathway in Cardiovascular Disease

The DDR pathways, particularly the CGAS-STING1 pathway, have been targeted, mostly through genetic and to a lesser degree through pharmacological interventions, in mouse models of cardiovascular diseases (Table 1). CGAS and IRF3 have emerged as the 2 most attractive targets, the former is a major CDSP and the initiator, and the latter is the effector of the active CGAS-STING1 pathway.

**Table 1:** Preclinical Studies (Mouse Models) Targeting the CGAS-STING1 Pathway

Condition	Target	Intervention	Phenotypic Effects
Lamin A-cardiomyopathy	CGAS (MB21D1)	Conditional deletion of *Mb21d1* gene in cardiac myocyte	Improved survival and cardiac function, reduced cell death and fibrosis.^70^
Myocardial infarction/injury	IRF3	Systemic deletion of *Irf3* gene	98% survival 2 wks after myocardial infarction and improved cardiac function.^35^
	IFNAR	Systemic deletion of *Ifnar* gene	100% survival 2 wks after myocardial infarction and improved cardiac function.^35^
		Neutralizing antibody	Improved survival and cardiac function postmyocardial infarction.^35^
	CGAS (MB21D1)	Systemic deletion of *Mb21d1* gene	83% survival 2 wks after myocardial infarction and improved cardiac function.^35^
	STING1	Systemic deletion of *Sting1* gene (Sting^gt/gt^)	No survival benefit.^35^
	CGAS	Deletion of CGAS in platelets	Attenuates platelet activation and myocardial ischemia/reperfusion injury. ^48^
	STING1	Pharmacological inhibition	No effect on survival upon permanent coronary ligation but improved left ventricular systolic function upon ischemia/reperfusion injury.^47^
Cardiac hypertrophy	CGAS	AAV9-shRNA targeting of CGAS	Attenuated pressure overload-induced cardiac hypertrophy, fibrosis, apoptosis, and cardiac dysfunction and improved survival.^38^
	STING1	C-176 (STING inhibitor)	Attenuated chronic kidney disease-induced cardiac hypertrophy.^59^
	STING1	Deletion of *Sting1* gene	Reverses TAC-induced cardiac remodeling and dysfunction in PINK1 deficient mice.^77^
Doxorubicin cardiotoxicity	ZBP1	Constitutive knockout of *Zbp1* gene	Improved cardiac function and reduced myocardial fibrosis.^37^
	STING1	Constitutive knockout of *Sting1* gene	Improved cardiac function and reduced myocardial fibrosis.^37^
	IFNAR	Constitutive knockout of *Ifnar* gene	Improved cardiac function and reduced myocardial fibrosis.^37^
	STING1	Global and endothelial cell-specific deletion of *Sting1* gene	Ameliorated doxorubicin cardiomyopathy.^36^
	CGAS	Systemic deletion of *Mb21d1* gene	Ameliorated doxorubicin cardiomyopathy.^36^
	IRF3	Systemic deletion of *Irf3* gene	Ameliorated doxorubicin cardiomyopathy.^36^
Aortic aneurysm and dissection	STING1	Systemic deletion of *Sting1* gene (Sting^gt/gt^)	Reduced aortic aneurysm and dissection in response to diet and angiotensin II and reduced MMP9 production.^44^
	TBK1	Pharmacological inhibition with Amlexanox	Prevented aortic degeneration and aneurysm/dissection development.^44^
	STING1	Myeloid cell-specific deletion of *Sting1*	Reduces AAA incidence and aortic rupture.^66^
	IFNAR	Myeloid cell-specific deletion of *Ifnar*	Reduces AAA incidence and aortic rupture.^66^
Hypertensive cardiac dysfunction	CGAS	Systemic deletion of CGAS	Improved cardiac dysfunction.^65^
Systemic arterial hypertension	CGAS	Intracisternal infusion of RU.521 (CGAS inhibitor)	Reduced myocardial fibrosis, cardiac dysfunction, and hypertrophy.^65^

CGAS = cyclic guanosine monophosphate–adenosine monophosphate synthase; IFNAR = Interferon α and β receptors; IRF3 = interferon regulatory factor 3; MB21D1 = Mab21 domain containing protein 1; STING1 = stimulator of interferon genes 1; TBK1 = TANK-binding kinase 1; ZBP1 = Z-DNA binding protein 1.

### Genetic interventions

Both systemic and myocyte-specific deletion of the *Irf3* gene in mice afford protective effects against cardiac remodeling and death postmyocardial infarction (coronary ligation).^34^^,^^35^ Deletion of the *Irf3* or *Ifnar* gene afforded a near complete survival postmyocardial infarction, a finding that, if replicated in independent experiments, would render IRF3 and its receptors as very attractive therapeutic targets in myocardial infarction.^35^

Multiple cell types are implicated in the activation of the type I interferon response postmyocardial injury, including cardiac macrophages, dendritic cells, and a subset of cardiac myocytes, as discussed earlier.^34^^,^^35^^,^^46^ The existing data mainly focuses on the nDNA, but the mtDNA is also likely to be involved, and its role in the activation of the interferon response and cardiac remodeling postmyocardial infarction also merits further investigation. Collectively, the data point to the prominent role of the IRF3 and its receptors in the pathogenesis of cardiac remodeling postmyocardial infarction.^35^^,^^37^ Both deletion of the *Irf3* and *Ifnar* genes, the latter encoding the interferon α and β receptor subunit 1, affords salubrious effects in mouse models of myocardial infarction and heart failure.^35^^,^^37^ Finally, IRF3 also has been targeted in the setting of other cardiovascular diseases but not in the context of DNA damage, and therefore, such studies are not discussed.

The beneficial effects of genetic targeting of the DDR pathways in heart failure have been demonstrated in mouse models of hereditary cardiomyopathies.^43^^,^^70^ Deletion of the *Mb21d1* (CGAS) gene, or the *Tp53* gene encoding TP53 protein, prolonged survival, improved cardiac function, attenuated cell death, and reduced myocardial fibrosis in mouse models of LMNA-cardiomyopathy.^43^^,^^70^ The beneficial effects were more pronounced upon the genetic inhibition of the CDSP pathways as opposed to the genetic blockade of the ATM-TP53 pathway.^43^^,^^70^ These findings, which show greater beneficial effects of blocking CDSP than the TP53 pathways, suggest the involvement of the mtDNA, likely in addition to that of the nDNA, in the activation of the CGAS-STING1 pathway, albeit it is not a direct comparison between the 2 interventions. Consistent with these findings, suppression of expression of CGAS in the heart upon the expression of a shRNA using adeno-associated viruses has been reported to improve cardiac function and attenuate myocardial fibrosis and cell death in mice.^38^

Cooperative interactions between CGAS and ZBP1 induce type I interferon response and activate the cell death programs in myocardial disease, including in doxorubicin-induced cardiomyopathy.^37^^,^^53^ Consequently, deletion of the *Zbp1*, *Ifnar1*, or *Sting1* gene attenuates doxorubicin-induced cardiac dysfunction and myocardial fibrosis.^37^ STING1, which is a hub molecule in the CDSP pathway, is also an attractive therapeutic target in cardiovascular conditions, including in mouse models of doxorubicin-induced cardiac dysfunction, myocardial infarction, and aortic aneurysm and dissection, among others.^35^^,^^37^^,^^44^ However, STING1's multiple functions, including its beneficial role in autophagy, raise some concerns about its attractiveness as a therapeutic target.^21^^,^^30^

### Pharmacological inhibition of the CGAS-STING1 pathway

Given the salubrious effects of genetic deletion of the key molecules involved in the CDSP pathway and the pathogenic role of the activation of the CGAS-STING1 pathways in various diseases, there is considerable interest in developing effective pharmacological inhibitors of this pathway for therapeutic gains. The existing data have largely focused on pharmacological activation and inhibition of this pathway, depending on the specific condition, in immunological diseases and cancer. The recent data showing the beneficial effects of the genetic blockade of the CDSP pathway in cardiovascular conditions await replication through the pharmacological inhibition of this pathway in cardiovascular diseases. Given the crucial role of the CDSPs in the innate immune response, systemic inhibition of the CDSP pathway might impart unexpected and fortuitous effects, including an increased risk of infection and possibly cancer. Therefore, the potential benefits of inhibiting the CDSP must be balanced against such adverse outcomes.

There are over a dozen experimental small molecules that are designed to inhibit CGAS and there are several activators and inhibitors of STING1, which are mainly being evaluated in immunological diseases and cancer.^71^, ^72^, ^73^ Concerning CGAS inhibitors, these agents either block the binding of CGAS to DNA, inhibit its catalytic function in generating cGAMP, or affect its activity through post-translational modifications. A notable example is sodium salicylate (aspirin), which directly acetylates CGAS, and inhibits its activity and the expression of type I interferon response genes.^74^ Thus far, the effects of these small molecules in cardiovascular diseases remain to be tested through carefully designed and long-term studies.

Cyclic GAMP, which activates STING1, is degraded by ecto-nucleotide pyrophosphatase/phosphodiesterases (ENPPs) such as ENPP1.^75^ Inhibition of ENPPs using a monoclonal antibody was found to protect the heart against cardiac remodeling and dysfunction in a mouse model of myocardial infarction by enhancing mitochondrial function and reducing cell death and fibrosis.^76^^,^^77^ These effects, however, are likely to be independent of the cytosolic cGAMP levels, because increased cytosolic cGAMP upon inhibition of ENPP1 would be expected to activate STING1 and impart deleterious effects on cardiac structure and function.

### Current progress and challenges

There is ample evidence, as discussed in the previous text, to support the release of self-DNA, both nuclear and mitochondrial DNA, to the cytosol in pathological conditions. One might speculate, based on the abundance of the data, that the presence of the cytoplasmic self-DNA is a ubiquitous finding in the pathological states and even likely in the physiological state and aging, the latter albeit at a lower level. Likewise, the data strongly supports the activation of the CDSP pathway in response to the release of self-DNA to the cytosol, as presented in the previous text. Furthermore, the bulk of the data but not all suggests that blocking the CDSP, at least through genetic deletion of the key genes in the pathway, imparts salubrious effects in cardiovascular diseases. However, despite these promising findings, there are several lingering concerns. To begin, there are some inconsistencies in the findings, and not all studies have shown beneficial effects. The discrepancies in part may reflect the shortcomings of the study designs, such as a lack of a proper control group, an inadequate sample size of the study, a paucity of evidence of the effectiveness of the intended intervention, as well as inadequate data analysis, biases in data acquisition and interpretation, and others. Despite these shortcomings and the presence of some discrepancies in the findings, by and large, the data suggests that the activation of the CDSP is pathogenic, and the genetic blockade of the pathway affords salubrious effects. Whether the findings of the genetic interventions would extend to pharmacological interventions designed to inhibit the key molecules in the CDSP pathways remains an empiric question that awaits careful testing, despite a few preliminary data providing favorable findings. Equally unclear is whether the long-term blockade of the CDSP pathways, whether through genetic or pharmacological interventions, as opposed to short-term interventions, would be innocuous as has been commonly observed in the short-term genetic studies. Considering that the activation of the CDSP pathway is designed to fight against the invading foreign DNA and impose cell death on the infected cells to prevent the propagation of the invading pathogen, the question remains as to whether the long-term blockade of the CDSP pathway, which typically would be necessary for chronic cardiovascular diseases, would also impart deleterious effects. It remains to be tested whether blocking the CDSP pathway would reduce the ability of the organism to fight against the invading pathogens. Hypothetically, it might be preferable to suppress the CDSP pathway and do so intermittently, as opposed to its complete blockade, to maintain the cell's ability to fight against the invading pathogen. Likewise, the preferred approach(es) to target the CDSP pathway and its specific component that affords the most effective and safe approach to counter the pathogenic role of its activation in cardiovascular disease remains to be settled. Considering the functional multiplicity of the protein components of the CDSP pathway, empiric data, obtained through carefully designed studies, would be necessary to address unexpected and fortuitous effects and resolve the nagging safety concerns. Whereas the activation of the CDSP pathway is best recognized in the immune cells, the existing data also support the activation of this pathway in nonimmune cells, including cardiac myocytes, endothelial cells, and others. These findings also raise the prospect for cell type-specific intervention to block the CDSP according to the pathogenic role of the cell types involved in the specific disease.

The initial reports of blocking the CDSP pathway in the model organisms, mainly mice, have shown favorable effects. However, as often is the case, the initial enthusiasm, often based on inadequately designed experiments and overinterpreted findings, is often subdued by the emergence of more realist data upon conduct of the well-designed controlled studies, bringing to the fore the complexity of the biological pathways. Moreover, as has been typical throughout the last few decades, the results of even solidly designed and executed studies in the model organisms typically do not extend to human patients, which is the ultimate objective of conducting the experiments. Thus, the road ahead is long and winding, and traversing it successfully begets conducting robust studies in the model organisms, interpreting the findings critically, and extracting novel insights, hoping that such knowledge will allow extending the findings to the care of patients with cardiovascular diseases.

## Concluding Remarks and Future Directions

The discovery of CGAS as a canonical sensor of cytosolic DNA and activation of the CGAS-STING1 pathway in mediating type I interferon response and the NFκB proinflammatory pathway have heightened the interest of the cardiovascular investigators in targeting these recently identified components of the innate immune response for therapeutic gains. The renewed interest in therapeutic targeting of the innate immune response is in accord with the existing data supporting the pathogenic role of the activation of the DDR pathways, particularly the CDSP pathway as well as the salubrious effects of the blockade of the DDR pathways in cardiovascular disease. The stage is set for further substantiating the pathogenic role of cytosolic nDNA and mtDNA in cardiovascular disease and the beneficial effects of the targeting of the DDR pathways for preventive and therapeutic gains through robust preclinical and large-animal studies followed by the extension of the positive findings to humans.

## Funding Support and Author Disclosures

This work was supported in part by the National Institutes of Health, National Heart, Lung, and Blood Institute Awards R01 HL151737, R01 HL132401, and R01AG082751 to Dr Marian, R01HL174481 to Dr Gurha, and American Heart Association Career Development Award 23CDA1053101 to Dr Rouhi.
